# P-POSSUM Falls Short: Predicting Morbidity in Ovarian Cancer (OC) Cytoreductive Surgery

**DOI:** 10.3390/cancers17213421

**Published:** 2025-10-24

**Authors:** Michail Sideris, Mark R. Brincat, Oleg Blyuss, Samuel George Oxley, Jacqueline Sia, Ashwin Kalra, Xia Wei, Caitlin T. Fierheller, Subhasheenee Ganesan, Rowan E. Miller, Fatima El-Khouly, Mevan Gooneratne, Tom Abbott, Ching Ling Pang, Parvesh Verma, Seema Shah, Alexandra Lawrence, Arjun Jeyarajah, Elly Brockbank, Saurabh Phadnis, James Dilley, Ranjit Manchanda

**Affiliations:** 1 Wolfson Institute of Population Health, Queen Mary University of London, London EC1M 6BQ, UK; m.sideris@qmul.ac.uk (M.S.); o.blyuss@qmul.ac.uk (O.B.); s.oxley@qmul.ac.uk (S.G.O.); j.sia@qmul.ac.uk (J.S.); a.kalra@qmul.ac.uk (A.K.); x.wei@qmul.ac.uk (X.W.); c.fierheller@qmul.ac.uk (C.T.F.); s.ganesan@qmul.ac.uk (S.G.); 2 Department of Gynaecological Oncology, Barts Health NHS Trust, Royal London Hospital, London E1 1BB, UK; m.brincat1@nhs.net (M.R.B.); rowan.miller2@nhs.net (R.E.M.); mevan.gooneratne@nhs.net (M.G.); tom.abbott@nhs.net (T.A.); c.pang@nhs.net (C.L.P.); parvesh.verma3@nhs.net (P.V.); seema.shah4@nhs.net (S.S.); alexandra.lawrence5@nhs.net (A.L.); arjun.jeyarajah@nhs.net (A.J.); elly.brockbank@nhs.net (E.B.); s.phadnis@nhs.net (S.P.); james.dilley@nhs.net (J.D.); 3 Department of Pediatrics and Pediatric Infectious Diseases, Institute of Child’s Health, Moscow 119991, Russia; 4 Department of Medical Oncology, University College London Hospital, 250 Euston Road, London NW1 2PB, UK; 5 Department of Medical Oncology, Barking, Havering and Redbridge University Hospitals NHS Trust, Romford RM1 2BA, UK; fatima.el-khouly@nhs.net; 6 Department of Health Services Research and Policy, London School of Hygiene & Tropical Medicine, London WC1H 9SH, UK; 7 MRC Clinical Trials Unit at UCL, Institute of Clinical Trials & Methodology, Faculty of Population Health Sciences, University College London, London WC1V 6LJ, UK

**Keywords:** ovarian cancer, cytoreductive surgery, P-POSSUM, surgical morbidity, morbidity prediction model

## Abstract

Objective: The P-POSSUM scale is widely used in predicting perioperative morbidity and mortality. Evidence on the performance of P-POSSUM in predicting outcomes after cytoreductive surgery (CRS) for ovarian cancer (OC) is limited. In this study, we assess how well P-POSSUM predicts morbidity in OC CRS and explore whether incorporating additional clinical variables can enhance its predictive accuracy. We retrospectively collected data on consecutive patients undergoing OC CRS within a tertiary gynaecologic oncology network. The collected information included demographic characteristics, P-POSSUM morbidity and mortality scores, Edmonton Frail Scale (EFS) scores, preoperative serum albumin levels, and observed 30-day postoperative morbidity and mortality, classified using the Clavien–Dindo (CD) scale. The predictive performance of P-POSSUM was evaluated using receiver operating characteristic (ROC) curves to calculate sensitivity and specificity. A stepwise regression analysis was then applied to identify additional variables that could improve model performance, incorporating preoperative covariates. The final model incorporated parameters chosen through bootstrap investigation of the model variability (stepAIC). Predicted versus observed morbidity was calibrated and performance compared between P-POSSUM and the final model. Results: Of 161 sequential OC patients, 95 (59%) underwent primary, 45 (28%) interval, and 21 (13%) delayed CRS. The mean age was 66.4 (95%CI: 60–75) and duration of surgery was 223 mins (95%CI: 142–279). Sixty-five (40.3%) patients had ≥1 postoperative complication. Two deaths were reported. Among the observed complications, 4 patients (6.1%) experienced CD4, 10 patients (15.3%) CD3, 38 patients (58.5%) CD2, and 11 patients (16.9%) CD1 events. The mean P-POSSUM-predicted morbidity and mortality were 59.5% (95%CI: 56.7–62.3%) and 5.86% (95%CI: 5.02–6.70%), respectively. The area under the curve (AUC) for P-POSSUM in predicting morbidity and mortality was 0.539 (*p* = 0.401) and 0.569 (*p* = 0.137), respectively. Given the small number of deaths, no robust conclusions regarding mortality are possible. EFS and BMI emerged as significant predictors of observed morbidity using a stepwise-model selection process. The AIC of this final model was 211.44. Our final model of PPOSSUM + EFS + BMI had AUC = 0.6551 (Delong’s Z = 1.8845, *p*-value = 0.05949). Conclusions: The P-POSSUM scale shows poor performance for predicting morbidity in OC CRS. New validated and accurate model(s) are necessary for predicting surgical morbidity. Our proposed model incorporates additional variables to improve P-POSSUM’s performance. This requires further development and validation.

## Simple Summary

We evaluate the performance of the P-POSSUM scale in predicting perioperative morbidity in women undergoing cytoreductive surgery (CRS) for ovarian cancer (OC). Data from 161 consecutive patients were retrospectively analysed, including demographics, frailty (Edmonton Frail Scale—EFS), preoperative albumin, and surgical outcomes. Postoperative morbidity occurred in 40.3% of patients, with most complications graded as Clavien–Dindo II. P-POSSUM significantly overestimated morbidity (predicted 59.5% vs. observed 40.3%) and demonstrated poor discrimination (AUC 0.54). Mortality prediction was also suboptimal, though the small number of deaths limited interpretation. Step-wise regression with bootstrapping identified EFS and BMI as additional significant predictors of morbidity. Incorporating these into a combined model (P-POSSUM + EFS + BMI) improved predictive accuracy (AUC 0.66), though these improvements did not reach statistical significance. Overall, P-POSSUM alone is inadequate for morbidity prediction in OC CRS. A revised model integrating frailty and BMI shows promise; however, it requires prospective validation.

## 1. Introduction

Cytoreductive surgery (CRS) for ovarian cancer (OC) remains a cornerstone intervention with a significant impact on both progression free and overall survival [1]. Complete macroscopic tumour resection during CRS leading to zero residual disease (R0) is the strongest prognostic variable associated with significantly improved survival [2]. To achieve this, CRS often encompasses complex four-quadrant radical surgery with multi-visceral resection. The DESKTOP trials support CRS for recurrent OC in select cases to improve survival [3]. This radical CRS is associated with a significant overall 35% (8% intraoperative and a 27% postoperative) complication rate [4]. Major surgical complications and long-term sequelae, especially those related to the bowel and urinary tract systems and lymphedema, as well as menopausal symptoms, have a notably significant impact on patients’ well-being [5]. This inherent perioperative risk and need for complex surgery has led to centralisation of care to subspecialised teams and high-volume centres, guided by governance models to support patient safety [6]. Low- and middle-income countries (LMICs) face added challenges for developing such services, which are integral to the ovarian cancer treatment pathway, as these depend heavily on the provision of high-quality anaesthetic (including high-dependency/intensive care) and at times multi-disciplinary surgical services [7].

Patients undergoing CRS are often elderly with increased frailty, poorer physiological reserves influenced by increased tumour burden, cancer-related symptoms, suboptimal nutrition, and chemotherapy-related effects [8,9]. An increased frailty index, poor performance status, and surgical complexity are associated with a higher risk for severe postoperative complications [10]. Residual tumour and low albumin levels are also associated with poorer survival [10]. Increased frailty and severe complications are associated with delays in commencing postoperative chemotherapy post CRS [11]. The cumulative impact of these factors underpins the necessity for a more comprehensive understanding of preoperative baseline risks and predicting surgical morbidity.

To this end, several predictive risk models have been developed to quantify the perioperative risks for surgical complications. Those models enable healthcare professionals to triage suitable (fit) patients for CRS, and facilitate informed discussions with patients and their relatives, while reducing the risk of litigation following unfortunate outcomes [12]. The Portsmouth-Physiology and Operative Severity Score for the Enumeration of Mortality and Morbidity (P-POSSUM) evolved from its predecessor, POSSUM, developed by Copeland et al. in 1991 [13,14]. These models were developed through an extensive examination of various physiological and surgical parameters, identifying key variables that significantly influenced postoperative outcomes. By integrating these factors into a scoring system, P-POSSUM aimed to serve as a tool for operative risk stratification. P-POSSUM was developed and validated on a dataset of general surgical interventions, excluding paediatric and day cases. Since the original publication of POSSUM, the score has been modified and further validated for a number of surgical and clinical scenarios, including colorectal surgery [15], hepatobiliary surgery [16,17], and vascular surgery [18].

P-POSSUM is widely used for anaesthetic preoperative assessment pathways for OC radical CRS across many UK cancer centres, including routinely in our North-East London Cancer Alliance (NELCA) Network. Patients’ clinical data are routinely used in P-POSSUM/SORT predictive models to predict individualised morbidity and mortality risks at preoperative high-risk anaesthetic and surgical assessment appointments as a dedicated/customised pathway for radical CRS. Predicted complication rates from these models influence clinical and shared patient decision making and surgical planning. However, adequate model validation data specifically for radical CRS in OC patients are lacking and their accuracy remains undetermined in this context. We aim to evaluate and validate the performance of P-POSSUM in predicting morbidity and mortality in OC CRS. Additionally, we develop a revised predictive model with improved performance using P-POSSUM and additional covariates.

## 2. Materials and Methods

We conducted a retrospective observational study including consecutive patients who underwent primary/interval (post cycle 3) or delayed (post cycle 6) CRS for OC between January 2019 and December 2022 across the NELCA Network. Data collected comprised demographics including ethnicity, age, cancer stage and histology, pre-op albumin, ASA score, BMI, and frailty assessment using ‘Edmonton Frail Scale (EFS)’ [19]. Predicted morbidity and mortality using P-POSSUM and SORT (mortality-only) scales were obtained from our ‘High-Risk Anaesthetics’ preoperative anaesthetic clinic assessment undertaken for all patients prior to CRS. We recorded observed morbidity and mortality data, including detailed breakdowns of complication numbers and severity (Clavien-Dindo (CD) Scale) [20], as well as length of stay (LOS) on the ward or High-Dependency Unit (HDU) and operating time.

### 2.1. Statistical Analysis

Inferential univariate statistics were used to summarise our cohort. ROCs were used to evaluate the performance of the P-POSSUM and SORT scales (sensitivity and specificity). Data were calibrated for expected versus observed morbidity and mortality. Selected preoperative covariates were incorporated into a stepwise regression model to improve P-POSSUM morbidity performance. We implemented a bootstrap procedure to investigate the variability of model selection under the *stepAIC()* stepwise algorithm from the *MASSpackage* in R Studio for windows version 4.5.1. AIC (Akaike Information Criterion) is a measure used to compare models based on their goodness of fit while penalising them for complexity, helping to select the most simple and informative model. Selected covariates were included in the final regression model and a revised receiver operating characteristic curve (ROC) was plotted to predict morbidity. All analyses were performed on R Studio v4.5.1 for Microsoft Windows 11.

### 2.2. Ethics

This study was part of a clinical audit; approval was obtained through our local governance process (Clinical Effectiveness Unit Registration Number 14032).

## 3. Results

### 3.1. Summary of Our Cohort

We included 161 consecutive patients with a mean age of 66.5 years (CI: 64.7–68.2). N = 95 (59%) underwent PDS, N = 45 (28%) IDS, and N = 21 (13%) DDS for OC. Table 1 summarises our cohort’s characteristic preoperative parameters including BMI, ASA, albumin, tumour stage and histology, along with predicted and observed morbidity (P-POSSUM) and mortality (P-POSSUM and SORT). The mean predicted morbidity and mortality using the P-POSSUM scale were 59.5% (CI: 56.7–62.26) and 5.9% (CI: 5.02–6.7); the mean predicted mortality using the SORT scale was 3.4% (CI: 0.18–26). There were 2 (1.24%) deaths, and N = 65 (40.4%) women had at least one observed complication of any CD scale.

### 3.2. Calibration of Observed vs. Predicted Morbidity for P-POSSUM

**Figure 1:**
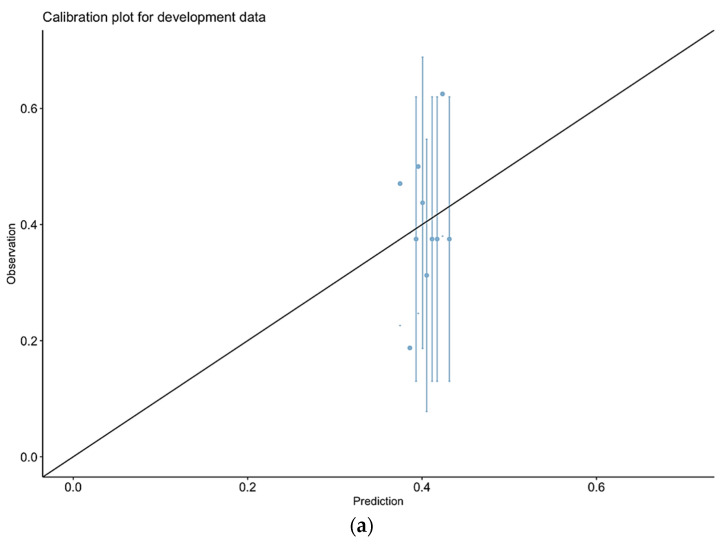

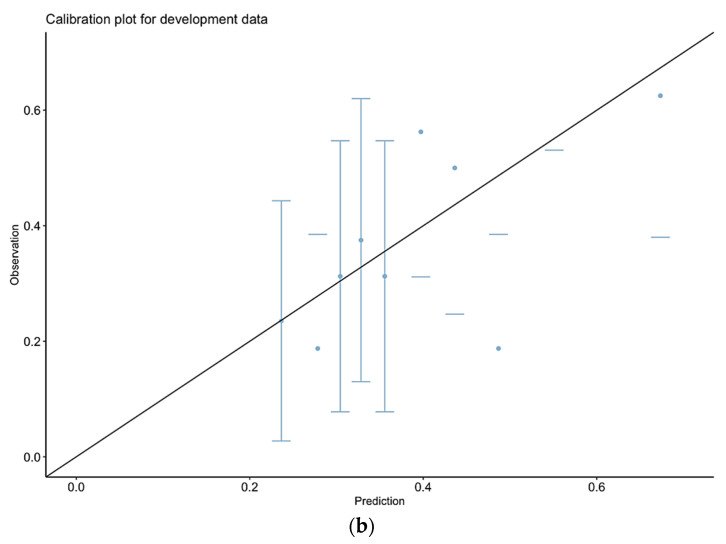
Calibration plots for prediction of morbidity for P-POSSUM alone (**b**) or combined model (**a**). P-POSSUM—(**a**); Final model—(**b**).

Figure 1a illustrates the calibration plot for predicted versus observed morbidity.

### 3.3. P-POSSUM Performance in Predicting Post-Operative Morbidity

**Figure 2:**
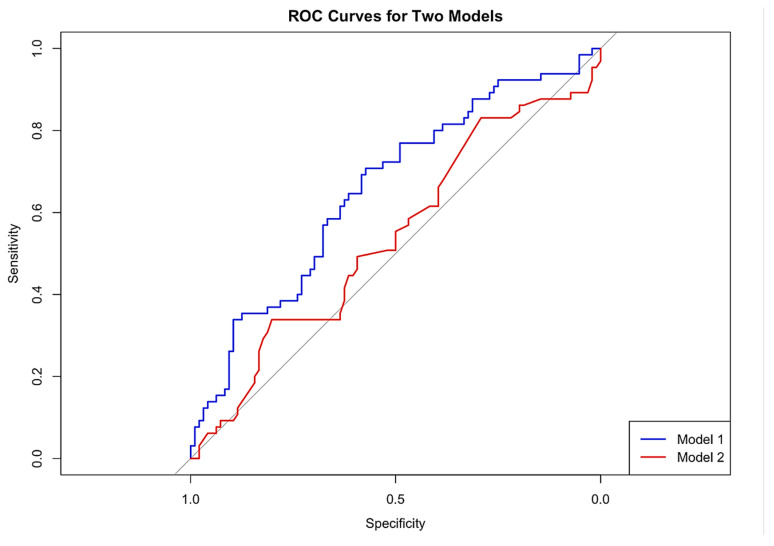
Model 1 = final model, Model 2 = P-POSSUM alone.

The area-under-the curve (AUC) for P-POSSUM alone is 0.5391 (Figure 2). Further exploratory analysis for P-POSSUM performance in predicting just major complications (CD3 and CD4) gave an AUC of 0.529.

### 3.4. Exploratory Stepwise Regression Analysis

Preoperative predictors including BMI, EFS, pre-op albumin, and the P-POSSUM scale were fed into a logistic regression model to predict morbidity. The model selection process was guided by the Akaike Information Criterion (AIC), using stepwise selection with the *stepAIC* function from the *MASSpackage* in R. The initial model included all four predictors with an AIC of 215.31 and a residual deviance of 205.31 (Appendix B). The stepwise model selection process identified EFS and BMI as the most significant predictors of observed morbidity; the AIC of this final model was 211.44.

### 3.5. Bootstrapping Stepwise Selection

To assess the robustness of model selection, we performed a bootstrap procedure with 2000 resamples (Appendix B). Across resamples, the stepwise procedure selected EFS and BMI in 96.25% and 93.19% of cases, respectively. In addition, EFS and BMI reached statistical significance (*p* < 0.05) in 54.20% and 52.12% of bootstrap models. P-POSSUM and preoperative albumin were less frequently selected (17.95% and 16.15%) and less often significant (35.93% and 31.89%). Based on these findings and clinical considerations, our final model included EFS, BMI, and P-POSSUM.

### 3.6. Comparisons of Final Model vs. P-POSSUM Alone for Predicting Morbidity

The area under the curve for our final model was 0.6551 compared to 0.5391 for P-POSSUM alone (Figure 2). Delong’s test to compare the ROCs for the two models showed (marginally) non-statistically significant difference (Z = 1.8845, *p*-value= 0.05949). Figure 2 includes the calibration plots for P-POSSUM and our final model. Exploratory analysis showed no difference between our final model (AUC = 0.53) and P-POSSUM in predicting major complications (CD3 and CD4) alone (DeLong’s test for two correlated ROC curves was Z = −0.21927, *p*-value = 0.8264).

### 3.7. Calibration of Observed vs. Predicted Morbidity for Our Final Model

Figure 1b presents the calibration plot for our final model, which shows improved alignment between predicted and observed morbidity, indicating better calibration compared to P-POSSUM alone. In both cases (Figure 1a,b), the large error bars indicate that there is considerable uncertainty in some of these observed frequencies.

### 3.8. Prediction of Mortality Using P-POSSUM and SORT Scales

Since we only had two deaths in our cohort, interpretation of these results is limited, and it is not possible to validate the outcome of mortality. ROCs with associated AUC and calibration plots are provided on Appendix C. The AUC for P-POSSUM scale mortality prediction is 0.616 and, for the SORT scale, 0.6211.

A review of studies reporting morbidity and mortality outcomes based on P-POSSUM outcomes highlighted 11 studies, which are presented in Appendix A.

## 4. Discussion

### 4.1. Summary of Main Results

We showed that P-POSSUM performed poorly specifically in the context of predicting morbidity for OC CRS, with the area under the curve being 0.5391. This is close to ‘random chance’. The P-POSSUM calibration plot (Figure 2) shows significant deviations from ideal calibration, particularly at higher predicted probabilities. This miscalibration further highlights the limitations of P-POSSUM for clinical decision making, underscoring the need for better-performing models which incorporate the specific patient, clinical, and procedural factors inherent to the OC CRS setting. The P-POSSUM and SORT scales appeared to overpredict deaths too, as we observed only two (1.24%) in our cohort. However, the number of events is too small to draw any meaningful conclusions.

There is limited data on validation of P-POSSUM for CRS not only for OC but also across gynaecological oncology surgery per se. While P-POSSUM has been a widely accepted tool in general surgical practice, it showed poor performance when applied to our cohort. Our cohort included two deaths, hence interpretation of the predictive value of the P-POSSUM or SORT mortality scale is limited.

Our new model, which incorporated EFS and BMI, improved the model’s performance (P-POSSUM + EFS + BMI), achieving an AUC of 0.655. This improvement neared statistical significance (*p* = 0.059), suggesting this may become significant with a larger dataset and highlighting the need for further prospective validation in a larger independent dataset. This underpins the value of including objective assessments of frailty (EFS) and BMI in preoperative risk assessment. The calibration plot of the final model indicates a closer alignment between predicted (expected) probabilities and observed outcomes across different risk strata. This improved calibration suggests that our revised model provides more reliable risk predictions, particularly at higher risk levels where accurate prediction is crucial for clinical decision making. Exploratory analysis showed no difference in predicting major complications (CD3 and CD4) alone between our final model and P-POSSUM, and the small numbers/sample size may have contributed to this.

Frailty refers to multisystem impairments that develop separately to the normal ageing process and has been recognised as a critical factor influencing postoperative outcomes [11,21]. Frail patients typically have reduced physiological reserves, making them more susceptible to postoperative morbidity, and frailty is associated with a challenging and protracted recovery period [21]. Our observation is supported by studies indicating that frailty assessments can enhance the predictive accuracy of surgical risk models [10,11]. Our findings reinforce the importance of incorporating frailty measures into preoperative evaluations to better stratify risk and tailor perioperative care.

BMI reflects a patient’s nutritional, metabolic, and fitness status [22,23]. Elevated BMI is part of the metabolic syndrome, which may include insulin resistance, with clear sequalae in postoperative healing and the recovery process. On the contrary, low BMI may indicate cachexia, with associated sarcopenia and low albumin levels [24,25]. Our study supports the inclusion of BMI as a straightforward and valuable metric in preoperative risk assessment.

### 4.2. Results in the Context of Published Literature

The P-POSSUM model was designed to estimate surgical risks predominantly in a general surgery setting. It has previously been evaluated across multiple surgical specialties (Appendix A). A meta-analysis [16] of 16 studies on hepatobiliary surgery highlighted that while POSSUM overpredicted postoperative morbidity, P-POSSUM was more accurate for predicting mortality. However, the authors suggested that both models require modifications for better applicability in hepatobiliary surgery.

A German study [26] on colorectal cancer (CRC) cases reported accurate predicted mortality within the 60–70% and 80–90% risk groups, with significant overprediction in the other lower risk groups. Similarly, the colorectal POSSUM (CR-POSSUM) model was found to overpredict mortality, reaching maximal accuracy in the 50–60% risk decile. Horzic et al. [27] and Tez et al. [28] both evaluated P-POSSUM and CR-POSSUM in colorectal cancer surgery, finding that P-POSSUM underpredicted mortality by 25%, whereas CR-POSSUM was slightly more accurate.

An Australian study [29] compared the performance of P-POSSUM with the POSSUM, NZRISK, and SORT scores in predicting 30-day mortality in general surgery. They found it to have high discrimination; however, there was a consistent tendency to overpredict this risk. The SORT scale was identified as the best performer in this comparison.

Mukherjee S et al. [30] investigated a cohort of gastrointestinal (GI) and gynaecological cancer cases who required HDU or ITU postoperative admission and concluded that P-POSSUM overpredicted morbidity, with a low correlation coefficient (0.24) between predicted morbidity and observed complications. Notably, patients with P-POSSUM scores of 60% and above experienced a higher incidence of major complications, highlighting the additional challenges encountered in this subgroup. Our findings are consistent with those of Das et al. [31], who concluded that P-POSSUM tends to overestimate the risk of mortality in gynaecological cancer surgeries in general, predicting a 7% mortality rate against an observed rate of only 2%. The same study reported an association in the overall operative (complexity) scores with morbidity (Spearmann’s rho 0.386, *p* < 0.001); however, their methodology was not designed to directly assess the performance of P-POSSUM.

Overall, while P-POSSUM and its variants offer valuable insights, their predictive accuracy varies significantly across different surgical contexts, underscoring the need for continual refinement and validation to evaluate their reliability and applicability.

SORT utilises patient health variables and information on the planned surgical procedure to estimate the risk of death solely within 30 days of surgery. In an external validation study for SORT, Wong et al. calculated the score for 475 hepatectomy procedures, reporting an AUROC of 0.82. In this cohort, SORT overpredicted mortality rates, particularly in patients with the lowest risk profiles [32]. Oakland et al. showed that, in patients undergoing major abdominal surgery, SORT performed well in patients with lower risk profiles, but underpredicted adverse outcomes in the higher risk group [33]. Unfortunately, our mortality data were too small to draw any meaningful inferences.

### 4.3. Strengths and Weaknesses

Our study is the first to evaluate P-POSSUM purely for OC CRS. Our data include all cases in a consecutive case series, diagnosed across a wide London region (cancer alliance) with an ethnically and socially diverse background, suggesting that our findings could be representative and applicable to broader UK clinical practice. Additionally, we employed rigorous statistical methodology for model evaluation and subsequent development, including stepwise regression and bootstrapping, to enhance the robustness of our results.

Nevertheless, our study has a number of limitations including its retrospective design, relatively small sample size, and the low number of mortality events, which particularly restricts the power of our mortality prediction analysis. Additionally, while we had access to the P-POSSUM scores for all patients from their clinical records, we lacked the raw data for each of the individual variables which were incorporated into each P-POSSUM assessment. Given our limited sample size, we could not further explore the effect of covariates including BMI. For instance, we were unable to comment on the impact of obesity or sarcopenia on postoperative morbidity. Adding albumin in conjunction to BMI did not significantly improve the model performance. Similarly focusing the analysis on FIGO stage IIIC-IV OC cases unfortunately did not return statistically significant results.

Furthermore, we were unable to capture data on the complications/impact of neoadjuvant chemotherapy (NACT) prior to surgery or evaluate its impact or sub-groups of IDS and PDS on peri/postoperative morbidity. The SUROVA trial concluded that OC cases who received NACT and underwent IDS had shorter hospital stays, reduced blood loss, fewer severe (CD Grade ≥ 3) postoperative adverse events, and lower mortality compared to those undergoing PDS [34]. Given sample size limitations, attempts to perform sensitivity analysis on advanced stage OC would underpower our results. Our model needs further prospective development and validation in a larger multi-centre dataset.

### 4.4. Implications for Practice and Future Research

Importantly, POSSUM/P-POSSUM were not developed from a dataset of patients undergoing OC CRS, and hence, unsurprisingly, the model performs poorly in this context. P-POSSUM does not capture the unique patient characteristics, clinical aspects of disease biology, and complexities, including multivisceral resections, associated with OC CRS. These elective operations differ substantially from those typically encountered in general surgery. In our study, the P-POSSUM AUC for morbidity prediction was 0.539, suggesting a poor ability to discriminate between patients who would experience postoperative complications and those who would not. In our view P-POSSUM should no longer be used in the clinical practice for predicting morbidity and mortality for OC CRS. This overestimation of risks could lead to unnecessary alarm, increased anxiety, and potentially inappropriate clinical decision making, which could adversely influence treatment outcomes. Overprediction of risks may also increase anxiety amongst patients more than is necessary. Our development of a new model highlights the need and ability to improve on P-POSSUM, which is currently being used in clinical practice. Additionally, the P-POSSUM scale provides an overall likelihood of experiencing ‘any complication’ without discriminating the severity of it (CD scale). This is a major limitation, particularly when providing counselling and planning for complex OC CRS.

There is a clear unmet need to develop more accurate and personalised operative risk prediction models for OC CRS. This is also needed for other complex gynaecological oncology surgical procedures. Arguably, different models are needed for different tumour types given the wide range and differences in the surgical procedures undertaken in these contexts. Models are likely to better perform and be better validated on the types of datasets that are used to create them. Newer statistical methodologies such as advanced machine learning (ML) techniques, which are able to leverage comprehensive raw data for development and validation, will improve predictive ability. When designing a new model, it would be important to include ‘traditional’ surgical risk factors such as age, BMI, and past medical history, as well as novel comprehensive raw data which have not been considered previously, i.e., tumour burden or surgical complexity. These data may include genetic or molecular data and imaging parameters which may more accurately predict surgical complexity. ML algorithms, particularly those employing deep learning and neural networks, can analyse these multifaceted big datasets to develop predictive models with improved accuracy. It also allows for continuous model improvement, incorporating new data and feedback from clinical outcomes to enhance predictive accuracy. National and international collaboration will be needed to progress this and ensure generalisability.

### 4.5. Sample Size Considerations and Validation of a Future Model

Assuming a prevalence of 0.5 for any complication, 0.2 for Clavien–Dindo Grade 3–4, and 0.02 for mortality, we provide sample size estimates below for AUCs of both 0.7 and 0.8 for these outcomes for a new prediction model. For each fixed prevalence and AUC value, we calculate two sample sizes and provide the largest values in Appendix D. To ensure accurate predictions and mitigate overfitting, we adopted the four-step framework proposed by Riley et al. [35]. Assuming inclusion of 25 predictor parameters, the minimum required sample size for model development was estimated for prevalences of 0.5, 0.2, and 0.02 as 1737, 2690, and 21,720, respectively, for AUC = 0.7 and 731, 1109, and 8430 for AUC = 0.8, respectively.

For external validation, we applied the sample size methodology described by Riley et al. [36], targeting precise estimation of both the observed-to-expected (O/E) ratio and the C-statistic. Assuming an anticipated O/E ratio near 1.0 and a 95% confidence interval width of 0.2, the required sample size was determined as the maximum of the two estimates. For prevalences of 0.5, 0.2, and 0.02, the sample sizes estimated were 416, 1538, and 18,839 for AUC = 0.7 and 385, 1538, and 18,839 for AUC = 0.8, respectively.

## 5. Conclusions

The P-POSSUM model has poor applicability in predicting morbidity and perhaps mortality for patients undergoing CRS for OC. Incorporating EFS and BMI into the predictive model can improve its predictive accuracy. This study underscores the need for specifically developed tailored risk assessment tools in CRS, paving the way for future studies to build on our findings. As healthcare continues to move towards personalised medicine, leveraging comprehensive datasets and advanced ML analytics will be essential in developing robust and reliable predictive tools. This is vital to improve decision making and surgical outcomes, enhance patient satisfaction, and ultimately lead to better overall health outcomes for women with OC.

## Figures and Tables

**Table 1:** Demographics.

**Mean age** (range), SD (years)	66.50 (27.00–88.00), *SD* = 11.3			
**Mean BMI** (range), SD (kg/m^2^)	27.93 (16.00–55.26), *SD* = 6.10			
**Mean pre-op albumin** (range), SD (g/dL)	40.48 (12.00–51.00), *SD* = 6.56			
**Stage at diagnosis** N (%)	**Stage 1**	**Stage 2**	**Stage 3**	**Stage 4**
	23 (14.3%)	19 (11.8%)	54 (33.5%)	65 (40.4%)
**ASA grade** N (%)	**ASA 1**	**ASA 2**	**ASA 3**	**ASA 4**
	7 (4.3%)	77 (47.8%)	74 (46.0%)	3 (1.9%)
**Histology** N (%)	**HGSOC**	**Clear Cell**	**Endometrioid**	**Mucinous**
	109 (67.7%)	12 (7.5%)	11 (5.8%)	10 (6.2%)
	**Low grade serous**	**Sarcoma**	**Mesonephric/Steroid**	**Granulosa**
	9 (5.6%)	6 (3.7%)	2 (1.2%)	2 (1.2%)
**Surgical outcomes**				
**Type of surgery** N (%)	**PDS** 95 (59%)	**IDS** 45 (28%)	**DDS** 21 (13%)	
**Duration of surgery** (mean, range, SD) (minutes)	223.71 (51.00–631.00), *SD* = 112.66			
**Overall length of stay** (mean, range, SD) (days)	9.22 (3.00–135.00), *SD* = 11.43			
**HDU length of stay** (mean, range, SD) (days)	3.82 (0.00–135.00), *SD* = 10.81			
**Morbidity and mortality scales**				
**P-POSSUM morbidity risk** mean (range), SD (%)	59.50 (8.80–98.10), *SD* = 17.84			
**P-POSSUM mortality risk** mean (range), SD (%)	5.87 (0.40–49.60), *SD* = 5.40			
**SORT mortality risk** mean (range), SD (%)	3.41 (00.18–26.00), *SD* = 3.32			
**Frailty assessment**				
**Edmonton Frail Scale** mean (range), SD (/max score 17)	3.44 (0.00–15.00), *SD* = 2.58			
**Morbidity/mortality**				
**Highest level of postoperative Clavien–Dindo complication** N (%)	**CD1**	**CD2**	**CD3**	**CD4**
	11 (6.8%)	38(23.6%)	10(6.2%)	4 (2.5%)
**Total complications per patient** N (%)	**0**	**1**	**2**	**3**
	96 (59.6%)	26 (16.1%)	22 (13.7%)	14 (8.7%)

Cohort characteristics summary (SD—standard deviation; HGSOC—high grade serous ovarian cancer; PDS—primary debulking surgery; IDS—interval debulking surgery; DDS—delayed debulking surgery; CD—Clavien–Dindo).

**Figure A1:**
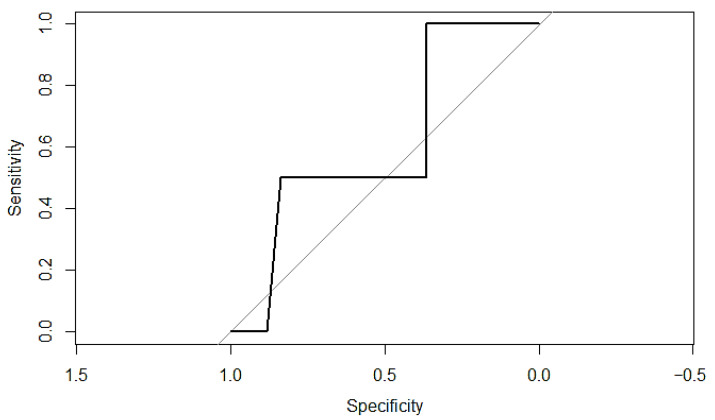
P-POSSUM mortality ROC.

**Figure A2:**
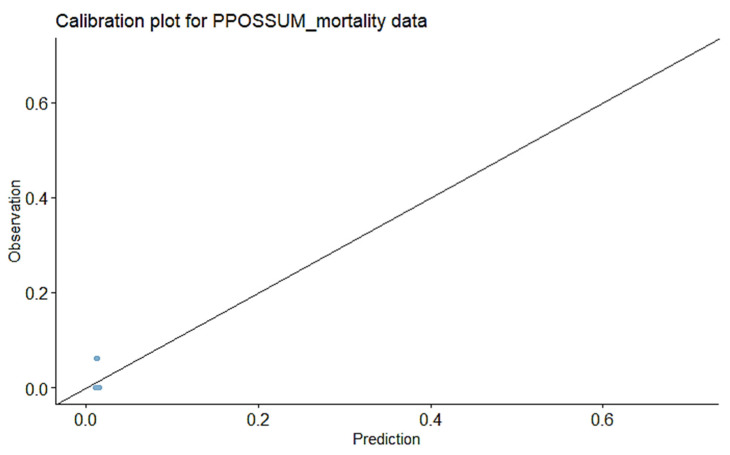
P-POSSUM mortality calibration plot.

**Figure A3:**
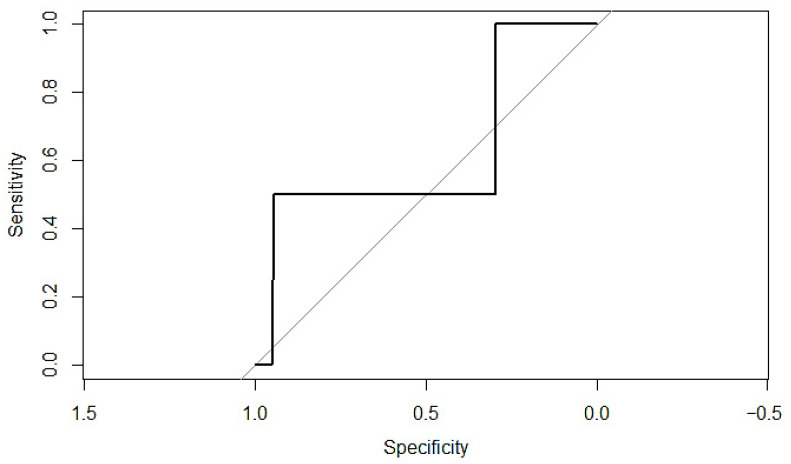
SORT mortality ROC.

**Figure A4:**
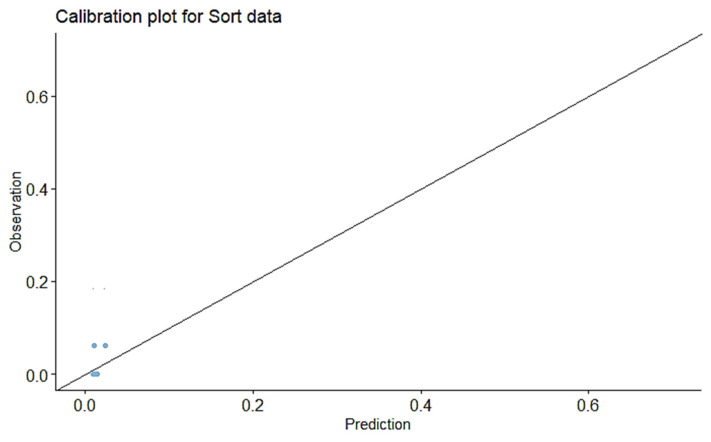
SORT mortality calibration plot.

## Data Availability

Dataset available on request from the authors.

## Appendix A. Search Strategy, Table Summary of Selected Studies

Search strategy:

(PPOSSUM OR POSSUM OR (Portsmouth-Physiological and Operative Severity Score for the enumeration of Mortality and Morbidity (P-POSSUM) OR P*POSSUM)) AND ((((surgical morbidity) OR (surgical mortality)) OR (surgical complications)) OR (clavien*dindo))

Selected studies:

**Table A1:** Review of studies discussing PPOSSUM scale application across several surgical specialities.

PMID	Author, Year, Country	Population (Sample Size)	Setting (Abdominal Procedures, e.g., GO)	Intervention = Scale Assessed, i.e., P-POSSUM/ POSSUM Morbidity/Mortality	AUC If Available (for Each Outcome)	Structured Summary (Conclusions)
38202180	Burtin et al., 2023 [26], Germany	485 patients	Colorectal cancer surgery	POSSUM, P-POSSUM, and CR-POSSUM morbidity and mortality	The P-POSSUM model demonstrated accurate mortality predictions in the 60–70% (O 1.01) and 80–90% (O 1.19) risk ranges but significantly overestimated mortality in other categories. Similarly, the CR-POSSUM model consistently overestimated mortality across all risk levels, with its highest accuracy observed in the 50–60% risk range, yielding an O ratio of 0.89.	POSSUM overpredicted postoperative morbidity. All three scoring systems considerably overpredicted in-hospital mortality. POSSUM score identified patients at risk of anastomotic leakage, sepsis, and return to theatre. The three scoring systems were deemed too imprecise for the estimation of perioperative complications and mortality of patients undergoing colorectal surgery. A revision of the scoring systems could increase their reliability in the clinical setting.
35706623	Mukherjee et al., 2022 [30], India	143 patients	Adults undergoing gastrointestinal and gynaecological cancer surgeries who postoperatively required admission to an intensive care unit or high dependency unit.	P-POSSUM morbidity	The correlation coefficient between the predicted morbidity and observed complication was 0.24.	P-POSSUM was not a reliable predictor of postoperative morbidity for patients undergoing major gynaecological and gastrointestinal surgeries for cancer in this institution. There was a significant incidence of major complications with P-POSSUM morbidity prediction score ≥ 60%, leading to the need for more stringent assessment and monitoring in that subgroup.
35042495	Valenzuela et al., 2022 [37], Colombia	350 patients. 89.1% of patients had no neoplastic diagnosis	Abdominal surgery	POSSUM and PPOSSUM morbidity and mortality		POSSUM scoring overestimated the risk of morbidity and mortality in patients with high-moderate risk, while the P-POSSUM score was a more accurate predictor of mortality risk.
37588588	Torlot et al., 2022 [29], Australia	31,153 patients	General surgery	30-day mortality using SORT, NZRISK, P-POSSUM; POSSUM)	AUC: SORT = 0.922 NZRISK = 0.909 P-POSSUM = 0.893 POSSUM = 0.881	All four risk scores showed high discrimination for 30-day mortality but consistently overpredicted risk. SORT was the best performing risk score. Categorising patients based on SORT into low, medium (80–90th percentile), and high risk (90–100th percentile) might guide future allocation of perioperative resources. No tools were sufficiently calibrated to support shared decision making based on absolute predictions of risk.
23435569	Chen et al., 2013 [16], China	Meta-analysis of 16 studies	Hepatobiliary surgery	P-POSSUM morbidity and mortality	Morbidity analysis: POSSUM O/E ratio of 0.78 [95%CI 0.68–0.88]. Mortality analysis: POSSUM 0.35 (95%CI 0.17–0.54) P-POSSUM 0.95 (95%CI 0.65–1.25).	POSSUM overpredicted postoperative morbidity after hepato-biliary-pancreatic surgery. Compared with POSSUM, P-POSSUM was more accurate for predicting postoperative mortality. Modifications to POSSUM and P-POSSUM are needed for audit in hepato-biliary-pancreatic surgery.
18025331	Horzic et al., 2007 [27], Croatia	120 patients	Colorectal cancer surgery	P-POSSUM and Cr-POSSUM mortality	AUC for P-POSSUM was 0.70 and for CR-POSSUM was 0.59.	The P-POSSUM system underpredicted mortality by 25%, with no significant difference between the predicted and observed values (*p* = 0.96). The observed to expected ratio for Cr-POSSUM was 1.11, with no significant difference between the observed and predicted values (*p* = 0.19). Both P-POSSUM and Cr-POSSUM perform well in predicting mortality after colorectal cancer surgery, but Cr-POSSUM is more accurate. There is a constant need for re-evaluation of existing and any new scoring systems outside original development and validation populations.
16914285	Das et al., 2006 [31], United Kingdom	468 patients	Gynaecological oncology patients	P-POSSUM morbidity and mortality		The P-POSSUM algorithm overestimates the risk of mortality for gynaecological oncology patients undergoing surgery. The P-POSSUM algorithm will require further adjustments prior to adoption for gynaecological cancer surgery as a risk adjusted surgical audit tool.
17103102	Tez et al., 2006 [28], Turkey	321 patients	Colorectal surgery	P-POSSUM and CR-POSSUM mortality		Overall, 22 deaths were observed. CR-POSSUM predicted 25 deaths (chi2 = 12.20, *p* = 0.13), and P-POSSUM predicted 29 deaths (chi2 =18.85, *p* = 0.002). ROC curve analysis revealed that CR-POSSUM has reasonable discriminatory power for mortality. These data suggest that CR-POSSUM may provide a better estimate of the risk of mortality for patients undergoing colorectal resection.
16421662	Ramkumar et al., 2006 [38], UK	347 patients	Colorectal surgery	POSSUM, P-POSSUM, and CR-POSSUM morbidity and mortality	POSSUM AUC 0.752. PPOSSUM AUC 0.749. CR-POSSUM AUC 0.781.	Colorectal POSSUM provides comparable prediction of mortality risk after colorectal resection compared with POSSUM and P-POSSUM.
15048745	Lam et al., 2004 [39], China	259 patients	Hepatectomies	POSSUM and P-POSSUM mortality		POSSUM system overpredicted mortality risk in patients who had major hepatectomy for hepatocellular carcinoma. P-POSSUM significantly predicted outcome. A modified disease-specific equation was derived which requires prospective testing.
15048756	Mohil et al., 2004 [40], India	120 patients	Emergency laparotomy	P-POSSUM morbidity and mortality	Observed-expected ratios: Linear regression POSSUM morbidity 0.68 POSSUM mortality 0.39 P-POSSUM mortality 0.66 Exponential analysis POSSUM morbidity 0.91 POSSUM mortality 0.62 P-POSSUM mortality 0.88.	If analysed exponentially, POSSUM is a good predictor of morbidity and mortality in patients undergoing emergency laparotomy. P-POSSUM predicts mortality equally well. Both equations may be used for risk-adjusted surgical audit of patients undergoing emergency laparotomy.

## Appendix B. StepAIC and Bootstrapping Parameter Selection Process for Regression Model

		 		
		**StepAIC**
		 
		Start: AIC=215.3
		Outcome=Complication(Y/N): Model parameters: EFS + PPOSSUM_MORBIDITY + Pre-op Albumin + BMI
		 
		                         Df Deviance    AIC
		- Pre-Op Albumin          1   205.38 213.38
		- PPOSSUM_MORBIDITY       1   205.40 213.40
		  <none>                      205.31 215.31
		- BMI                     1   207.39 215.39
		- EFS                     1   215.84 223.84
		 
		Step:  AIC=213.38
		Outcome=Complication(Y/N): Model parameters: EFS + PPOSSUM_MORBIDITY +BMI
		 
		                         Df Deviance    AIC
		- PPOSSUM_MORBIDITY       1   205.44 211.44
		  <none>                      205.38 213.38
		- BMI                     1   207.44 213.44
		- Edmonton_Frailty_Score  1   216.26 222.26
		 
		Step:  AIC=211.44
		Outcome=Complication(Y/N): Model parameters: EFS + BMI
		 
		                         Df Deviance    AIC
		  <none>                      205.44 211.44	
		- BMI                     1   207.50 211.50
		- Edmonton_Frailty_Score  1   216.48 220.48
		 
		Call:  glm(formula = NumberComplicationBinomia ~ Edmonton_Frailty_Score + 
		    BMI, family = “binomial”, data = dppossum)
		 
		Coefficients:
		           (Intercept)  Edmonton_Frailty_Score                     BMI  
		              -0.06565                 0.22262                -0.03965
	     
		Degrees of Freedom: 160 Total (i.e. Null);  158 Residual
		Null Deviance:	    217.2 
		Residual Deviance: 205.4 	AIC: 211.4
		 
		 
		**Summary of Bootstrapping the ‘stepAIC()’ procedure for**
		 
		Call:
		glm(formula = NumberComplicationBinomia ~ Edmonton_Frailty_Score + 
		    PPOSSUM_MORBIDITY + Pre_Op_Albumin + BMI, family = “binomial”, 
		    data = dppossum)
		 
		Bootstrap samples: 2000
		Direction: backward
		Penalty: 2 * df
		 
		Covariates selected
		                         (%)
		Edmonton_Frailty_Score 96.25
		BMI                    54.20
		PPOSSUM_MORBIDITY      17.95
		Pre_Op_Albumin         16.15
		Null                    1.60
		 
		Coefficients Sign
		                        + (%) - (%)
		Edmonton_Frailty_Score 100.00  0.00
		Pre_Op_Albumin          31.27 68.73
		PPOSSUM_MORBIDITY       25.63 74.37
		BMI                      0.65 99.35
		 
		Stat Significance
		                         (%)
		Edmonton_Frailty_Score 93.19
		BMI                    52.12
		PPOSSUM_MORBIDITY      35.93
		Pre_Op_Albumin         31.89
		 
		 
		The stepAIC() for the original data-set gave
		 
		Call:  glm(formula = NumberComplicationBinomia ~ Edmonton_Frailty_Score + 
		    BMI, family = “binomial”, data = dppossum)
		 
		Coefficients:
		           (Intercept)  Edmonton_Frailty_Score                     BMI  
	                  -0.06565                 0.22262                -0.03965 
		 
		Degrees of Freedom: 160 Total (i.e. Null);  158 Residual
		Null Deviance:	    217.2 
		Residual Deviance: 205.4 	AIC: 211.4
		 
		Stepwise Model Path 
		Analysis of Deviance Table
		 
		Initial Model:
		NumberComplicationBinomia ~ Edmonton_Frailty_Score + PPOSSUM_MORBIDITY + 
		    Pre_Op_Albumin + BMI
		 
		Final Model:
		NumberComplicationBinomia ~ Edmonton_Frailty_Score + BMI
		 
		 
		                 Step Df   Deviance Resid. Df Resid. Dev      AIC
		1                                         156   205.3046 215.3046
		2    - Pre_Op_Albumin  1 0.07089549       157   205.3755 213.3755
		3 - PPOSSUM_MORBIDITY  1 0.06897021       158   205.4444 211.4444
		 
		

## Appendix D. Sample Size Considerations and Validation for Future Model

We assume a prevalence of 0.5 for any complication, 0.2 for Clavien–Dindo Grade 3–4, and 0.02 for mortality, and provide sample size estimates for AUC of both 0.7 and 0.8 for these outcomes. For each fixed prevalence and AUC value, we calculate two sample sizes and provide the largest values below.

### Model Development

We have taken steps to ensure that our models have accurate predictions and avoid overfitting by using the four-step approach outlined by Riley et al. [35]. Assuming inclusion of 25 predictor parameters, the table below shows the minimum number of participants required to achieve the recommended sample size for model development, depending on outcome prevalence and expected AUC values.

		**Prevalence of Outcome**		
		**0.5**	**0.2**	**0.02**
AUC	0.7	1737	2690	21,720
	0.8	731	1109	8430

Model validation: Taking the approach of Riley et al. [36] and bearing in mind that the predictive model has not yet been developed, we calculated the required sample size for the precise estimation of observed divided by expected cases and the C-statistic.

For the O/E statistic, we anticipate a value close to 1.0 and aim for a 95% confidence interval width of 0.2 to ensure adequate precision and demonstrate good calibration-in-the-large.

The table below presents the target sample size for external validation, calculated as the maximum of the two requirements—based on O/E precision and AUC estimation.

		**Prevalence of Outcome**		
		**0.5**	**0.2**	**0.02**
AUC	0.7	416	1538	18,839
	0.8	385	1538	18,839
